# Case Report: Anesthetic Management and Electrical Cardiometry as Intensive Hemodynamic Monitoring During Cheiloplasty in an Infant With Enzyme-Replaced Pompe Disease and Preserved Preoperative Cardiac Function

**DOI:** 10.3389/fped.2021.729824

**Published:** 2021-12-13

**Authors:** Meng-Chen Liu, Ming-Tse Wang, Philip Kuo-Ting Chen, Dau-Ming Niu, Yu-Hsuan Fan Chiang, Ming-Hui Hsieh, Hsiao-Chien Tsai

**Affiliations:** ^1^Department of Anesthesiology, Taipei Medical University Hospital, Taipei Medical University, Taipei, Taiwan; ^2^Department of Plastic and Reconstructive Surgery, Taipei Medical University Hospital, Taipei, Taiwan; ^3^Department of Pediatrics, Taipei Veterans General Hospital, Taipei, Taiwan; ^4^School of Medicine, National Yang Ming Chiao Tung University, Taipei, Taiwan; ^5^Graduate Institute of Medical Sciences, College of Medicine, Taipei Medical University, Taipei, Taiwan; ^6^Dianthus MFM Clinic Taoyuan, Dianthus MFM Center, Taipei, Taiwan

**Keywords:** Pompe disease, cardiomyopathy, hypotonia, anesthesia, impedance cardiography, pulse wave analysis

## Abstract

**Introduction:** Pompe disease is caused by deficiency of the lysosomal enzyme acid α-glucosidase, which results in cardiac and muscular complications that can jeopardize perioperative outcomes. We report a 4-month-old infant with Pompe disease receiving cheiloplasty under general anesthesia with the aid of peripheral nerve blocks and intensive hemodynamic monitoring.

**Case Description:** This case report describes a 4-month-old full-term Taiwanese female infant who presented with left unilateral cleft lip and palate in the prenatal examination. She was diagnosed with infantile-onset Pompe disease after acidic α-glucosidase (GAA) gene sequencing. She also received enzyme replacement therapy (ERT) 15 days after birth and regular ERT every other week. Cheiloplasty was performed under general anesthesia uneventfully, and peripheral nerve blocks were adopted for analgesia. Intensive hemodynamic monitoring using electrical cardiometry technology (ICON^®^) and pulse contour analysis (FloTrac system) were applied during the operation. No adverse effects were observed, and the wound healed well. Therefore, the patient was discharged 4 days after surgery.

**Conclusion:** With the availability of ERT, severe organ dysfunction in infantile-onset Pompe disease patients is no longer common. However, moderate cardiac depression can still occur while increasing inspiratory pressure and deepening the anesthesia level despite a normal preoperative echocardiogram report. Therefore, careful, gradual titration is desirable. Furthermore, electrical cardiometry can detect hemodynamic changes more instantaneously and reliably than pulse contour analysis. In addition, we suggest taking advantage of the peripheral nerve block as a part of balanced anesthesia to alleviate the cardiac suppression caused by general anesthesia.

## Introduction

Pompe disease, an autosomal recessive disorder, is caused by deficiency of the lysosomal enzyme acid α-glucosidase, which metabolizes glycogen in lysosomes (1, 2). Glycogen that is not able to be metabolized is stored in the organs, especially the heart, skeletal muscle, and liver, which causes hypertrophic cardiomyopathy, hypotonia, and hepatomegaly (3). Patients with infantile-onset Pompe disease may appear normal but exhibit hypotonia and feeding difficulties later. They may also present with atelectasis and respiratory distress due to the compression of the bronchi by an enlarged heart (4). In the past, the life expectancy of these infants was typically <1 year because of cardiorespiratory failure. Sudden death due to ventricular outflow tract obstruction was previously reported (3). Although the generalized use of enzyme replacement therapy (ERT) with recombinant acid α-glucosidase protein, which can improve cardiac function and increase survival rate (5), has reduced these deaths dramatically (2), patients receiving general anesthesia are still at high risk due to the higher incidence of cardiac arrest and respiratory insufficiency.

Here, we report a female infant with infantile-onset Pompe disease who underwent cheiloplasty under general anesthesia and peripheral nerve blocks. In addition, we applied intensive hemodynamic monitoring using electrical cardiometry technology (ICON^®^) and pulse contour analysis (FloTrac system) intraoperatively. This study was approved by the Taipei Medical University Joint Institutional Review Board (N202104013). We have obtained the written informed consent from her parents. De-identification was done according to Health Insurance Portability and Accountability Act (HIPAA) Privacy Rule safe harbor method.

## Case Description

**Figure 1:**
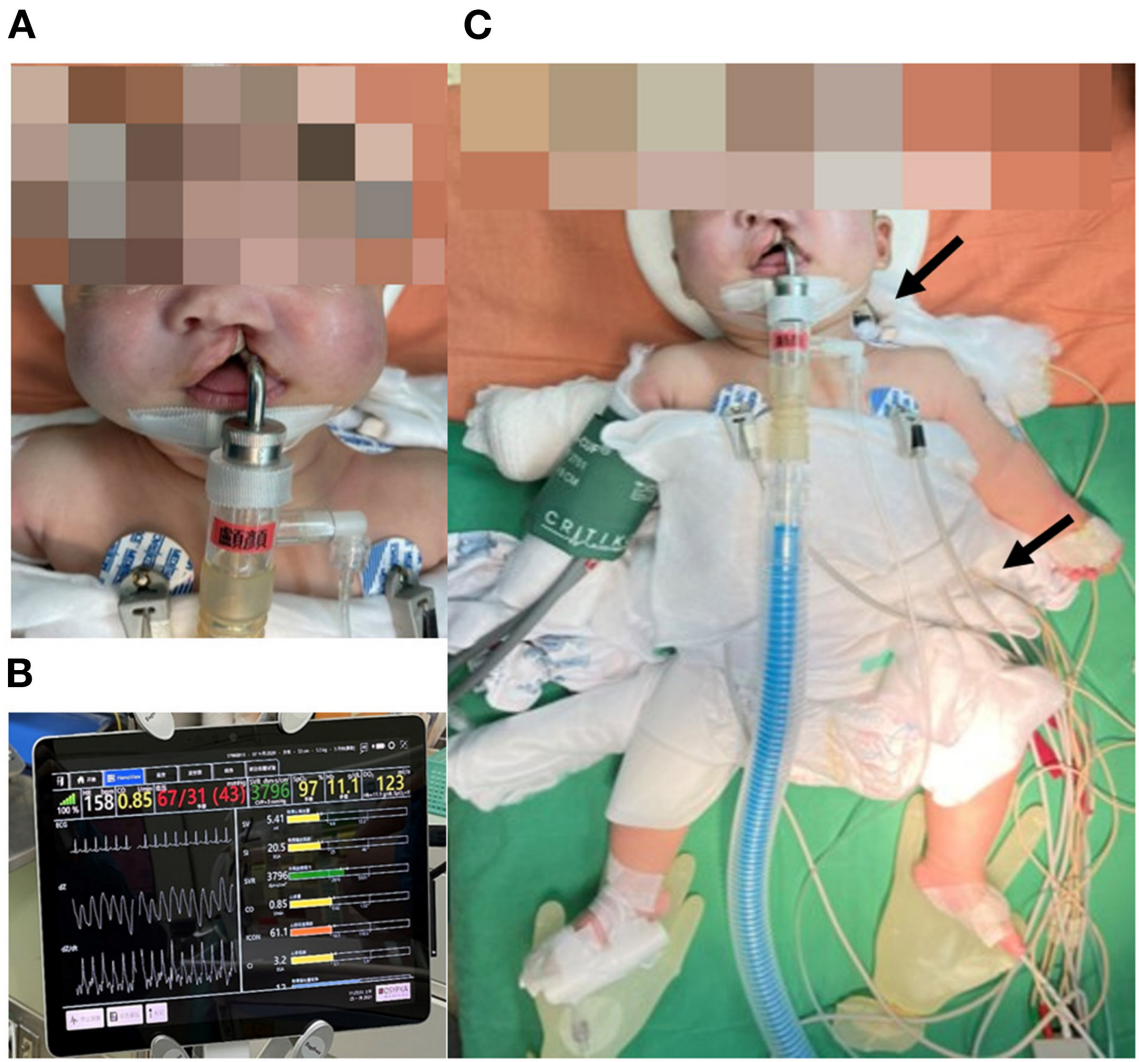
**(A)** The patient presented with left complete cleft lip and palate. Hypotonia and macroglossia were not observed, which may be related to regular ERT beginning at 15 days old. **(B)** Electrical Cardiometry Technology (ICON^®^) calculated and provided instant SV, SI, SVR, CO, ICON, and CI during the operation. **(C)** ICON^®^ sensors were located at the left side of the neck and thorax. Arrows represent the sites of electrode patches. ERT, enzyme replacement therapy; SV, stroke volume; SI, stroke volume index; SVR, systemic vascular resistance; CO, cardiac output; ICON, index of contractility; CI, cardiac index.

This case report describes a 4-month-old full-term Taiwanese female infant weighing 5.5 kg. She was born through spontaneous delivery, and her birth weight was 2,645 g, which was small for the gestational age of 38 weeks. Cleft lip and palate were observed in the patient in the prenatal examination. After birth, she presented symptoms of mild hypotonia without dysphagia or dysarthria. Occasional shortness of breath was noticed (Figure 1A). Laboratory data revealed abnormally high aspartate aminotransferase (AST), alanine aminotransferase (ALT), and creatinine kinase (CK), which were 125 U/L (normal range: <32 U/L), 48 U/L (normal range: <33 U/L), and 872 U/L (normal range: 26–192 U/L), respectively. The enzyme activity test of GAA was 0.04 μM/h (normal range: >0.80 μM/h). X-ray showed cardiomegaly (Supplementary Figure 1A). Left ventricular mass index (LVMI) was 105.49 g/m^2.7^, which was over the 95th percentile for her age (<6 months old) (6, 7). GAA gene sequencing was performed, and the patient had two pathogenic heterozygous mutations, c.424_440del17 (p.Ser142Leufs^*^29) and c.1935C>A (p.Asp645Glu). One heterogenous pseudodeficiency mutation, p.Gly576Ser, was also noticed. Her cross-reactive immunological material (CRIM) status was positive linked to c.1935C>A (p.Asp645Glu). All above findings indicated the diagnosis of Pompe disease. She received regular ERT at an initial dosage of 20 mg/kg intravenous infusion every other week beginning at 15 days old until 2 days before surgery. Preoperative echocardiography revealed preserved LV systolic function with minimal tricuspid regurgitation. Patent ductus arteriosus (PDA) and patent foramen ovale (PFO) were closed. LVMI was 65.5 g/m^2.7^, which was in the 75th percentile for her age (<6 months old) (6, 7). Her AST, ALT, and CK were decreased to 56, 33, and 160 U/L before the surgery, respectively.

The patient was admitted for scheduled cheiloplasty due to left complete unilateral cleft lip and palate. Upon arriving in the operating room, pulse oximetry, and electrical cardiometry technology (ICON^®^ Osypka Medical GmbH, Berlin, Germany) (Figures 1B,C) were applied before induction. Intravenous induction was performed with thiamylal 30 mg (5.4 mg/kg), cisatracurium 1 mg (0.18 mg/kg), dexamethasone 1 mg (0.18 mg/kg), and atropine 0.05 mg (0.01 mg/kg). Subsequently, a non-invasive blood pressure cuff and electrocardiography were applied. Intubation was successfully performed with Pentax AWS-s100 (PENTAX Medical, Hamburg, Germany; video-assisted laryngoscope) on the first attempt. Additionally, we placed a radial arterial line and FloTrac system (Edwards Lifesciences, Irvine, CA, USA) for hemodynamic monitoring. Peripheral nerve blocks with 0.4125% ropivacaine was performed as a part of combined anesthesia and for perioperative analgesia, including bilateral infraorbital nerve block (1.4 mL on each side), bilateral external nasal nerve block (0.5 mL on each side), and nasopalatine nerve block (0.1 mL). Anesthesia was maintained with sevoflurane 1.8–3.1% and 49–65% oxygen concentration in the air. The initial ventilator setting was inspiratory pressure (P_Insp_): 13 cm H_2_O, positive end-expiratory pressure (PEEP): 0 cm H_2_O, respiratory rate (RR): 30 breaths per minute, and inspiratory-to-expiratory time (I:E ratio): 1:1.5. Our goal was to maintain mean arterial pressure (MAP) above 45 mmHg and heart rate (HR) between 90 and 160 bpm. We focused on five parameters of ICON^®^: index of contractility (ICON), systolic time ratio (STR), thoracic fluid content (TFC), stroke volume variation (SVV), and systemic vascular resistance (SVR). We focused on three parameters of FloTrac: cardiac output (CO), SVV, and SVR. We compared CO, SVV, and SVR between these two hemodynamic monitors (Table 1). Due to the age limitation (8), the data from FloTrac deviated from the physiological range and were too inaccurate to reflect the clinical condition.

**Table 1:** Comparison of hemodynamic parameters between electrical cardiometry technology (ICON) and pulse contour analysis (FloTrac).

**Time after anesthesia started (min)**	**NIBP (mmHg)**	**ABP (mmHg)**	^*^ICON CO(L/min)	^*^ICON SVR (dyn·s/cm^**5**^)	^*^ICON SVV (%)	^**^FloTrac CO (L/min)	^**^FloTrac SVR (dyn·s/cm^**5**^)	^**^FloTrac SVV (%)
45	58/30 (42)	59/28 (39)	0.79	3,842	10	14.9	152	10.3
75	63/34 (47)	66/33 (46)	0.8	3,838	8	15.7	180	21.1
105	63/32 (45)	63/32 (44)	0.81	3,993	9	15.6	174	8.8
135	63/32 (45)	66/32 (44)	0.82	4,013	7	15.6	176	9.6
165	63/31 (44)	67/31 (43)	0.83	3,596	7	15.9	167	6.8
195	62/31 (47)	68/31 (44)	0.84	3,861	8	16.0	173	10.1

*NIBP, non-invasive blood pressure; ABP, arterial blood pressure; CO, cardiac output (normal range: 0.82–1.52 L/min); SVR, systemic vascular resistance (normal range: 2,870–5,331 dyn·s/cm^5^), SVV, stroke volume variation (normal range: 5–15%). NIBP and ABP are manifested as systolic blood pressure/diastolic blood pressure (mean arterial pressure)*.

* *ICON: electrical cardiometry technology*.

** *FloTrac: pulse contour analysis*.

During early maintenance, we titrated the inhalational gas concentration from 0.7 to 1.0 minimal alveolar concentration (MAC) before the skin incision, and we observed that the hemodynamic parameters on ICON^®^ changed accordingly. ICON and SVR dropped gradually in 1 min, whereas CO increased. However, 3 min later, the decline in ICON and SVR was significant. CO remained at the same level, causing a decrease in arterial blood pressure. Therefore, we cautiously decreased the inhalational gas concentration. Detailed changes in ICON, SVR, and CO are displayed in Supplementary Figure 2. Fifty minutes after anesthesia induction, we changed the ventilator setting and increased P_Insp_ from 13 to 15 cm H_2_O. Arterial blood pressure dropped soon after. This combined with the observation that SVV was occasionally above the normal range suggested fluid deficiency; therefore, we prescribed bolus normal saline (3 mL) with no apparent improvement. The ventilator setting was adjusted back to the initial setting, and the inhalational gas concentration was slowly titrated to 0.6 MAC. Eighty minutes after anesthesia induction, we also increased the normal saline infusion rate, as SVV was above the normal range and TFC was low; however, MAP was stable and in the normal range. No additional analgesic agent was prescribed during maintenance. At the end of surgery, we prescribed neostigmine 0.125 mg and glycopyrrolate 0.025 mg as neuromuscular blocking agent reverse medication before extubation. Extubation was uneventful with normal respiratory function. She was discharged 4 days later without adverse events.

After 3 months of follow-up, the patient was able to roll over, lie prone on the forearms, and reach out for objects, and she had a social smile. However, she could not maintain a sitting position unassisted, indicating slower development potentially than other infants of the same age. In addition, mild hypotonia was still observed compared with other infants. The girl infant also underwent another surgery in our hospital several months later. Unlike the previous surgery, she was more tolerant of inhalational gas concentration titration, which may represent improved cardiac function under regular ERT. After receiving ERT treatment for half a year, her anti-alglucosidase-α antibody revealed positive. Therefore, hydrocortisone was added in the following ERT courses. Her anti-alglucosidase alfa antibody will be followed in the next few months to see if additional immunosuppressive agents are needed.

## Discussion

This case presented several unique aspects. First, the surgery was complex. The entire cheiloplasty procedure took approximately 4 h to complete. Furthermore, no study has described Pompe disease patients receiving cheiloplasty before. In addition, resolving surgical site bleeding and avoiding coughing or straining during emergence from anesthesia and extubation may be difficult in this surgery, and Pompe disease could aggravate these difficulties. Second, we used ICON^®^ as an intensive hemodynamic monitoring method. Few facilities will or are able to perform stress test for cardiac function assessment for infants. As mentioned in our study, these patients might have a normal static cardiac echocardiography report, but are not normal enough to face stress from surgery and general anesthesia. In previous studies, Sanders et al. (9) and Lotfy et al. (10) had suggested ICON^®^ to be a useful trend monitor. Also, with its safe and easy applicable method and comprehensive hemodynamic parameters, it turns out to be a practical hemodynamic monitor. Additionally, a randomized controlled trial (10) recruited 42 pediatric patients who underwent hepatoportoenterostomy, and CO reliability was compared across electrical bioimpedance cardiometer, ICON^®^, and transoesophageal Doppler (TED), and the results showed that ICON^®^ use was effective intraoperatively. However, due to inadequate sample sizes, more comparative studies are required to provide accurate data and evidence in infants. Third, peripheral nerve blocks were employed as a part of combined anesthesia to provide ideal analgesic effects. Utilizing peripheral nerve block in patients undergoing cleft lip and palate can prevent the patients from complications of respiratory depression and airway obstruction, which is induced by opioid (11, 12). Also, the patients are allowed to wake up pain free, and closure of the suture lines can be maintained (13, 14). In this case, impaired liver function was noted since birth which may result in poor opioid metabolism function. Also, decreased dosage of opioid was suggested by Racca et al. in concern of negative effect to cardiac and respiratory systems (15). The use of peripheral nerve blocks can provide opioid free anesthesia for Pompe disease patients thus create a safe practice. On the other hand, potential risks of peripheral nerve blocks include nerves, vessels injury and local anesthetics overdose (16).

In Pompe disease patients, the diagnostic challenge is that many diseases share similar symptoms and signs of Pompe disease. Diseases such as hypothyroidism, congenital muscular dystrophy, and other glycogen storage diseases, are needed to be differentiated. And the final diagnosis of Pompe disease was made due to laboratory data, gene sequence, and the organs being influenced (cardiac, liver, and muscle) (1).

Previously reported cases of infantile-onset Pompe disease patients who received general anesthesia were listed in Table 2. We performed a database search of PubMed^®^, using keywords of Pompe disease and anesthesia. Reference lists were also manually searched to include all the related studies. In these studies, most underwent a short procedure. Thirteen patients underwent central venous catheter insertion or muscle biopsy, and the remaining three patients underwent elective intubation, bronchoscopy, and bilateral inguinal hernia repair. Only 2 of the 16 patients received ERT, but bradycardia with wide QRS complexes and non-sustained ventricular tachycardia were still reported after induction and during the early phase of maintenance anesthesia, respectively. Among the 14 patients without ERT, 10 had complications such as bradycardia, torsade de pointes, ventricular fibrillation, and cardiac arrest. None of these patients involved intensive intraoperative hemodynamic monitoring methods.

**Table 2:** Summary of reported cases of infantile-onset Pompe disease patients who underwent general anesthesia.

**References**	**Age**	**LV mass index (g/m^**2**^)**	**ERT (+/——)**	**Procedure**	**Agents**	**Complications**
DeSena et al. (17)	3 m	N/A	**——**	Central venous catheter insertion	Sevoflurane, vecuronium	Ventricular fibrillation after vecuronium infusion
Wang et al. (18)	23 m	125	**+** (every other week from age 6 m to 17 m)	Elective intubation	Thiopental, succinylcholine, vecuronium	Bradycardia with wide QRS complexes and variable atrioventricular conduction after induction agent infusion
	2 y	76.8	**+**	Bronchoscopy, stomaplasty	Ketamine, sevoflurane, succinylcholine	Paroxysmal supraventricular tachycardia followed by nonsustained ventricular tachycardia under 2.4% sevoflurane (5 min after induction)
	8 m	704	**——**	Central venous catheter insertion	Propofol, fentanyl	Bradycardia and ventricular fibrillation after intubation
	14 d	59.3	**——**	Central venous catheter insertion	Propofol	Bradycardia (−21% to −31% from baseline) after propofol infusion
	4 m	191	**——**	Central venous catheter insertion, muscle biopsy	Sevoflurane, propofol, 40% nitrous oxide	Bradycardia, desaturation, and ventricular fibrillation after propofol infusion for maintenance
	5 m	446	**——**	Central venous catheter insertion, skin biopsy	Sevoflurane, propofol, nitrous oxide, rocuronium	Torsade de pointes VT on 2% sevoflurane in nitrous oxygen and oxygen maintenance (16 min after induction)
	2 m	253 (examined after this episode)	**——**	Bilateral inguinal hernia repair	Sevoflurane	Ventricular fibrillation after sevoflurane induction
	2 m	233	**——**	Muscle biopsy	Sevoflurane, nitrous oxide	Ventricular fibrillation and ventricular tachycardia after sevoflurane and nitrous oxygen induction
	8 m	363	**——**	Muscle biopsy, percutaneous gastrostomy, tunneled venous catheter placement	Etomidate, fentanyl, rocuronium	Hypotension and ventricular fibrillation under sevoflurane and nitrous oxide (14 min after induction)
Ing et al. (19)	5.2 ± 3 m	366	**——**	Central venous catheter insertion, muscle biopsy	Thiopental, sevoflurane, fentanyl, rocuronium	None
	5.2 ± 3 m	191	**——**	Central venous catheter insertion, muscle biopsy	Sevoflurane, nitrous oxide, propofol, rocuronium	Cardiac arrest under continuous maintenance infusion of propofol and 40% nitrous oxide in oxygen (shortly after induction)
	5.2 ± 3 m	240	**——**	Central venous catheter insertion, muscle biopsy	Sevoflurane, nitrous oxide, fentanyl, rocuronium	None
	5.2 ± 3 m	362	**——**	Central venous catheter insertion, muscle biopsy	Ketamine, nitrous oxide, sevoflurane, fentanyl, rocuronium	None
	5.2 ± 3 m	221	**——**	Central venous catheter insertion, muscle biopsy	Ketamine, nitrous oxide, fentanyl, rocuronium	None
McFarlane and Soni (4)	5 m	N/A	**——**	Hickman line insertion, marrow aspiration, liver biopsy, muscle biopsy	Nitrous oxide, halothane 2%, suxamethonium	Bradycardia and cardiac arrest after increasing halothane concentration and intubation

*LV, left ventricular; ERT, enzyme replacement therapy; N/A, data not found in the literature; y, years of age; m, months of age; d, days of age*.

The cause of arrhythmia of infantile-onset Pompe disease patients may be due to the intracardiac accumulation of glycogen inducing progressive hypertrophic cardiomyopathy and diastolic dysfunction. Therefore, venous return is obstructed and cardiac output decreases. Besides, certain anesthetics cause significant decrease in systemic vascular resistance or diastolic blood pressure, which result in decreased coronary perfusion and arrhythmia (18). Early diagnosis and enzyme replacement therapy resulted in inapparent cardiac dysfunction in our case, which prevented arrhythmia from occurring. During the whole procedure, no arrhythmia episode was presented in our case. However, mild TR in echocardiography and lower tolerance to anesthetics intraoperatively were still observed. Other concern in intubation was due to macroglossia in Pompe disease patients, but this phenomenon was not obvious in our case. With the help of video-assisted laryngoscope, intubation underwent smoothly. Regarding to atony in Pompe disease patients, we reduced the dose of muscle relaxant and also contacted intensive care unit for the risk of confronting difficult extubation. Finally, no related problem was confronted.

It might be possible for the Pompe disease girl infant to receive the surgery later, after receiving more courses of enzyme replacement therapy. However, many studies suggested to perform primary cleft lip repair between the ages of 3 and 6 months since it is proper timing, allows subsequent treatments to perform and also for better establishing lip competence (20, 21). Concerns of early surgical intervention in high-risk patients may be raised; however, with early enzyme replacement therapy in our case, cardiac and other organ functions seem to be tolerable to surgical stress and anesthetics.

Thiamylal, atropine, and cisatracurium were administered as induction agents and initially maintained under 2.2% sevoflurane. The selected hypnotic was thiamylal, which reduces SVR but increases the heart rate and is theoretically able to maintain CO. However, the use of barbiturates as intravenous induction agents is uncommon in the literature. Cisatracurium was selected as the neuromuscular blocking agent, as it is metabolized through Hoffman elimination and is thus unrelated to liver and renal function. Suxamethonium was not considered due to the associated risks of rhabdomyolysis and hyperkalemia in Pompe disease patients (19). Pompe disease, as a kind of metabolic myopathy, safe anesthesia in suspected myopathy suggested by Trevisan et al. (22) should be considered due to the risk of malignant hyperthermia. Although few events had been reported (23), additional attention should be paid to the changes of body temperature and end-tidal carbon dioxide (EtCO_2_) if halogenated inhalational anesthetics was chosen. After anesthesia induction, we utilized peripheral nerve blocks as additional analgesia. Only two studies have reported on infantile-onset Pompe disease patients who underwent muscle biopsy using peripheral nerve blocks but were without endotracheal intubation (16, 24). In our case, the patient received cheiloplasty, which requires general anesthesia. Sevoflurane is generally used in children with Pompe disease due to its advantage of rapid emergence. However, it can also simultaneously decrease SVR and myocardium contractility, thereby decreasing coronary perfusion pressure (17). Therefore, with the help of peripheral nerve blocks, the concentration of sevoflurane can be decreased, lowering the risk of inadequate coronary perfusion and arrhythmia.

**Figure 2:**
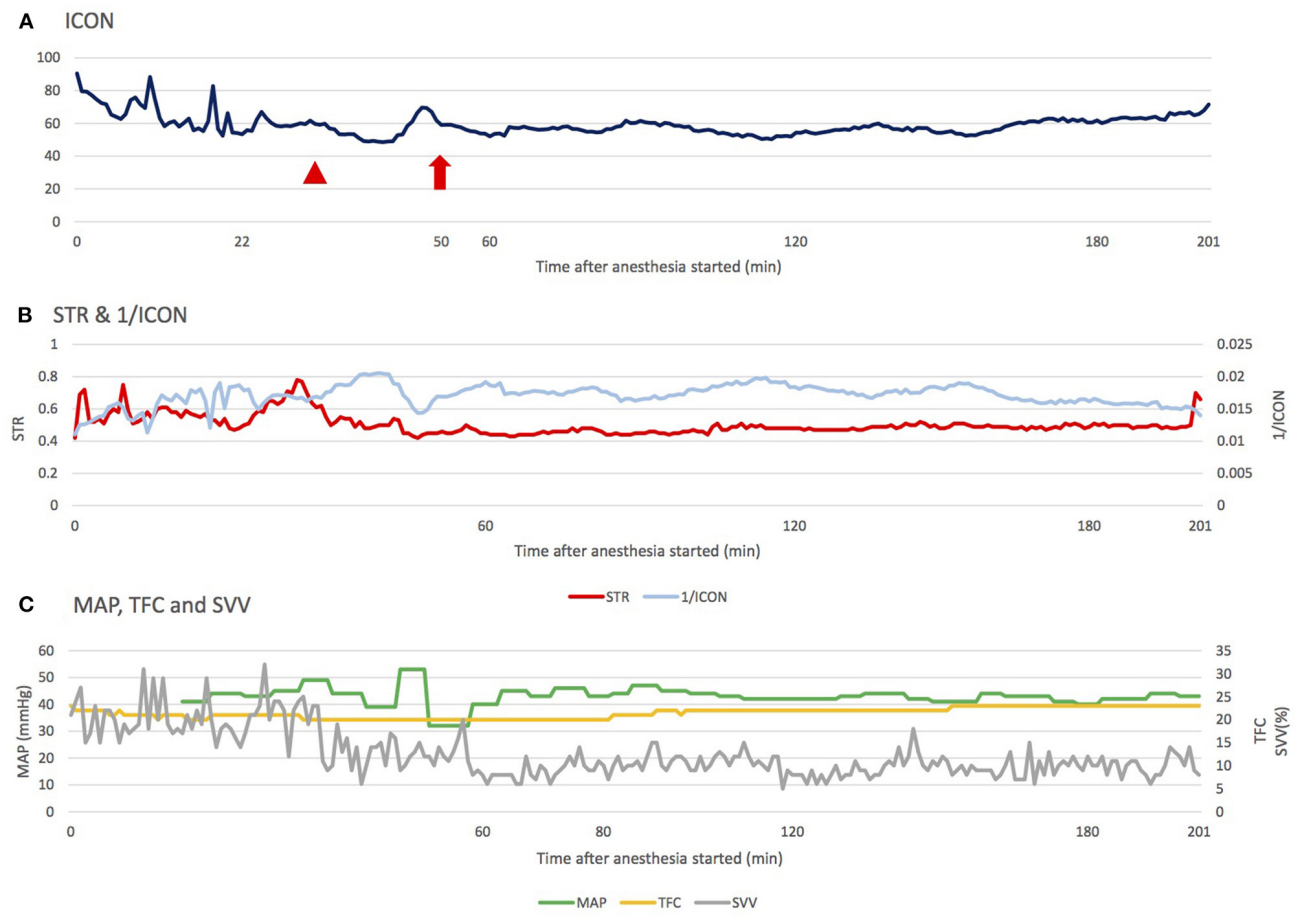
The changes of ICON, STR, MAP, TFC, and SVV during the operation. **(A)** During maintenance, ICON was mainly affected by the setting of inhalational agent concentration and inspiratory pressure in the ventilator. The arrowhead indicates the increased sevoflurane concentration before incision; the arrow indicates the alteration of ventilator setting: P_Insp_ from 11 to 13 cm H_2_O, respiratory rate from 30 to 28 breaths per minute (normal range of ICON: 45.0–75.0). **(B)** Changes in STR and 1/ICON were compatible, and STR seemed to react earlier, providing anesthesiologists a faster reference to adjust the anesthesia. **(C)** Fluid is another factor influencing perfusion. Regardless of the trend of change in TFC and SVV, MAP was able to compensate in a relatively stable range (Normal range of TFC: 25–35, SVV: 5−15%). ICON, index of contractility; STR, systolic time ratio; TFC, thoracic fluid content; MAP, mean arterial pressure; P_Insp_, inspiratory pressure.

We further analyzed the data of ICON, ventilator settings and the sevoflurane concentration using the Pearson correlation coefficient. A negative correlation between ICON and peak inspiratory pressure was observed (*R* = −0.212, *p* = 0.0026), while a positive correlation between ICON and inhalational gas concentration was noted (*R* = 0.2732, *p* < 0.0001). However, Figure 2A demonstrates that an increased inhalational gas concentration resulted in a drop of ICON value instead of a positive elevation. The positive correlation between ICON and inhalational gas concentration should be interpreted carefully. Because when icon decreased, and blood pressure consequently dropped, we tended to adjust the inhalational gas to a lower concentration. The positive correlation might be explained by our management rather than reflecting the actual relationship between these 2 parameters. Generally, an increase in peak inspiratory pressure and inhalational gas concentration would induce a drop in ICON; however, the titration of inhalational gas preserved ICON and CO at first. Later, an acute drop in ICON occurred, accompanied by a compensatory decrease in SVR and an increase in CO. Although sevoflurane is thought to be a safe anesthetic in Pompe disease patients, we should still keep concentrations low to prevent hypotension (18). One case report concluded that the increased risk of arrhythmia in patients with infantile-onset Pompe disease is likely related to a simultaneous decrease in SVR and myocardium contractility, decreasing coronary artery perfusion pressure (17). According to Shekerdemian et al. (25), positive pressure ventilation has been proved to reduce cardiac output because of reduced right ventricular filling. During inspiration phase of positive pressure ventilation, intrathoracic pressure increased results in elevated right atrium pressure, and eventually forms a decreased right ventricular filling and cardiac output. What's more, pediatric patients have a lower chest wall compliance compared to adult patients, so more intrathoracic pressure is needed to expand the same volume of chest cage in pediatric patients, which contributing to greater degree of cardiac output reduction. This phenomenon was also observed clinically by Gullberg et al. (26, 27) that the decreased mean airway pressure would increase cardiac output in neonates and infants. Therefore, in addition to minimizing the risk at induction and early maintenance related to anesthetic agents, extra attention must be focused on setting a suitable anesthetic depth and inspiratory pressure. We did not use electroencephalography (EEG) or alternative monitors such as entropy to evaluate her central nervous system function. This is because those monitors would interfere with the surgeon and contaminated the surgical field. No neurologist's visit was arranged preoperatively or postoperatively due to absence of newly onset neurological symptoms. However, the sequential performance of EEGs before and after the head and neck surgery could be taken into consideration as in cardiac surgery to exclude the potential embolus or small cerebral stroke in infant (28).

We also noticed a correlation between two contractility parameters in ICON^®^: STR and ICON. STR equates to the pre-ejection period (PEP) divided by the left ventricular ejection time (LVET). Theoretically, low STR correlates with high ejection fraction. ICON represents the acceleration of the blood in the aorta. After analysis, we found that STR was related to ICON. Moreover, STR could react faster than ICON when a large variation occurred (Figure 2B; to exhibit the same trend of changes, the data is presented as 1/ICON and STR). A possible explanation for this phenomenon is that STR is related to ejection fraction (EF), but ICON is related to blood speed and acceleration. Hence, once cardiac suppression occurs, STR will reflect this event faster than ICON. However, the accuracy of STR is dependent on adequate fluid status in patients. In circumstances of inadequate fluid status, ICON may still be more reliable.

Fluid is another factor influencing perfusion, and we monitored fluid status through the SVV and TFC parameters of ICON^®^ in this case. Mahmoud et al. (29) reported that TFC could be used to guide the fluid removal rate and amount in patients undergoing hemodialysis. The results showed that compared with the hemodynamically stable group, the hypotension group had lower TFC, indicating that they were hypovolemic. In our case, considering that SVV was more than the normal range and that TFC was lower initially, hypovolemia was potentially present. However, the infant's MAP could be compensated within a relatively stable range, which indicates that this infant's fluid supplement could not be guided by mean arterial pressure (Figure 2C).

Once Pompe disease is suspected, genetic screening, laboratory tests, electrocardiography, and image surveys should be conducted immediately. ERT should also be arranged as soon as the diagnosis has been made. Recent studies have revealed that with ERT intervention to prevent hypertrophic cardiomyopathy and a thorough preoperative evaluation, anesthesia management is much safer in infantile-onset Pompe disease patients (2, 17). A previous study reported a 13-year-old girl with juvenile Pompe disease who received ERT and underwent kyphoscoliosis corrective surgery (23). Fluid maintenance involved 450 mL of blood and 2.3 L of a lactated ringer under central venous pressure guidance, and the estimated blood loss was 1.1 L, which seemed to be acceptable in the general population. However, the patient experienced pleural effusion after surgery. One review article also noticed different types of arrhythmias occurred in some infantile-onset Pompe disease patients receiving ERT (30). Therefore, despite adequate ERT, mild degeneration in cardiac function can still occur. In our case, a similar condition was recorded. The patient was more sensitive to sevoflurane and changes in peak inspiratory pressure compared with other children, as a higher concentration of sevoflurane and a higher peak inspiratory pressure resulted in an apparent drop in ICON (Figure 2A) and, consequently, stroke volume index (SI). Otherwise, even in the era of ERT, there were still some limitations in treating Pompe disease, such as great expense, immune response due to high amounts of exogenous enzyme, progression of muscle weakness even responding well to ERT initially (30), limited effects to central nervous system due to blood brain barrier (31), and white matter abnormalities/ventricular enlargement in brain MRI (32). Some alternative therapies have been developed, like gene therapy (33, 34), enzyme enhancement therapy (35), and substrate reduction therapy (36). Further studies were required to determine the benefits of these treatments in decreasing perioperative risks for Pompe disease.

Our study has some limitations. First, we lacked strong evidence to clarify and support the accuracy of ICON^®^ intraoperatively in infants. More comparative studies may be needed to prove its use and validity in infants. Second, ICON^®^ depends on the theory of electrical bioimpedance cardiometry, which is interfered by electrocautery. In our case, ~10% of the data were of low quality and therefore excluded. Third, since the perioperative complication rate has dropped dramatically after the spread of ERT, cases with cardiac complications which we mentioned above were not fully comparable with current cases.

## Conclusion

The recent consensus suggests that early diagnosis and treatment with ERT can improve hemodynamic stability. However, with regular ERT, lower tolerance to anesthetic agents was still noted in this case. Peripheral nerve blocks can reduce the required dose of analgesic agents and make the processes of surgery and emergence safer and faster. For infants with existing cardiac pathophysiologic changes, ICON^®^ may be considered, as the trend of hemodynamic parameters can facilitate the advanced identification of potential problems, especially STR. Future studies should focus on ICON^®^ in infant perioperative monitoring to validate the safety and multiple parameters in this non-invasive hemodynamic monitoring method.

## Data Availability Statement

The raw data presented in the study are deposited in figshare repository. doi: 10.6084/m9.figshare.16955032.

## Ethics Statement

The studies involving human participants were reviewed and approved by Taipei Medical University-Joint Institutional Review Board. Written informed consent to participate in this study was provided by the participants' legal guardian/next of kin.

## Author Contributions

M-CL and H-CT had full access to all of the data in the study and take responsibility for the integrity of the data and the accuracy of the data analysis. D-MN helped us in acquisition of relevant data. All authors made substantial contributions to the study conception and acquisition of data, or analysis and interpretation of data, involved in drafting the article or revising it critically for important intellectual content, and approved the final version to be published.

## Conflict of Interest

The authors declare that the research was conducted in the absence of any commercial or financial relationships that could be construed as a potential conflict of interest.

## Publisher's Note

All claims expressed in this article are solely those of the authors and do not necessarily represent those of their affiliated organizations, or those of the publisher, the editors and the reviewers. Any product that may be evaluated in this article, or claim that may be made by its manufacturer, is not guaranteed or endorsed by the publisher.

## Supplementary Material

Supplementary Figure 1 **(A)** The girl infant's X-ray at 16 days old, which revealed cardiomegaly. **(B)** The electrocardiography of the girl infant at 15 days old, which revealed sinus tachycardia compared with other infants at the same age. Heart rate: 188 beats per minute; RR interval: 339 milliseconds (ms); PR interval: 56 ms; QRS duration: 99 ms; QT interval: 255 ms.

Supplementary Figure 2 Inhalational gas concentration was titrated from 0.7 to 1.0 MAC before skin incision (red arrows indicate the time of titration). ICON and SVR dropped gradually in 1 min, and CO increased. However, ICON and SVR dropped significantly 3 min later. By contrast, CO remained at the same level. MAC, minimal alveolar concentration; ICON, index of contractility; SVR, systemic vascular resistance; CO, cardiac output.
