# Supplementary figures and images for: Case Report: Anesthetic Management and Electrical Cardiometry as Intensive Hemodynamic Monitoring During Cheiloplasty in an Infant With Enzyme-Replaced Pompe Disease and Preserved Preoperative Cardiac Function
Source: Front Pediatr. 2021 Dec 13;9:729824. doi: 10.3389/fped.2021.729824 (PMC8710755; doi:10.3389/fped.2021.729824)

A

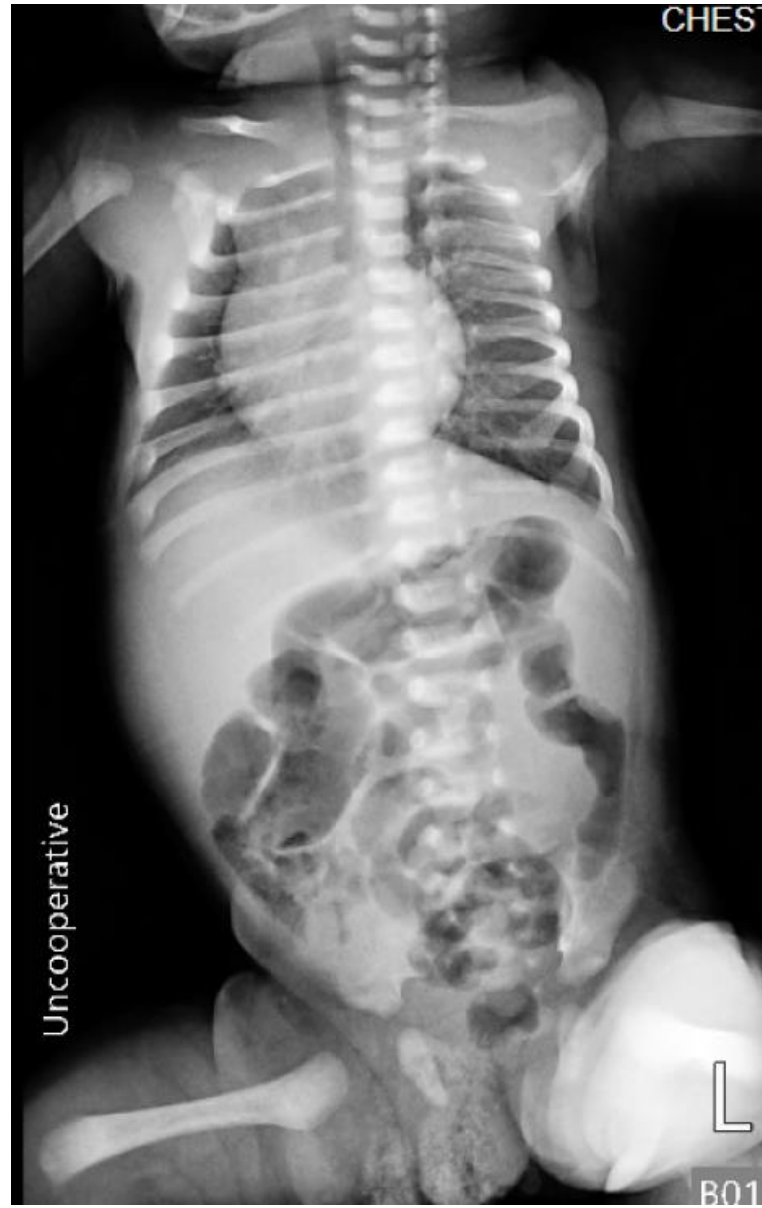

B

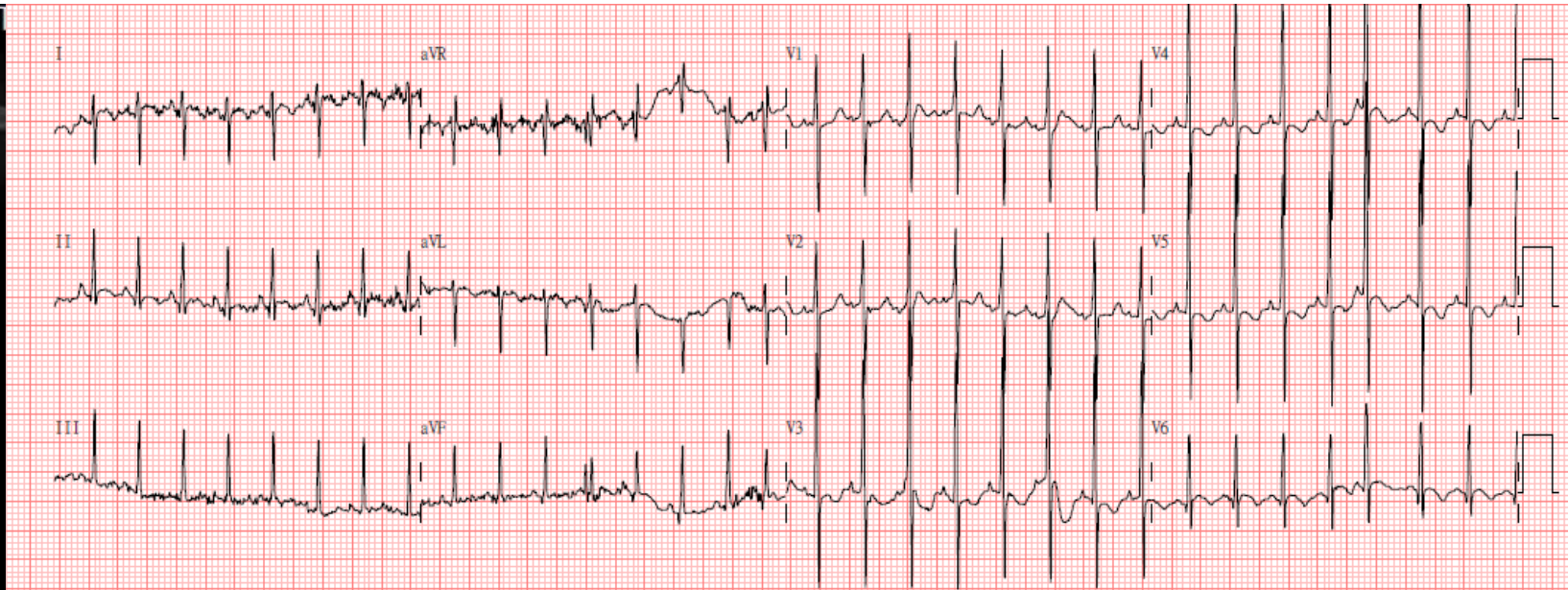

Supplement: Supplementary Figure 1 — (A) The girl infant's X-ray at 16 days old, which revealed cardiomegaly. (B) The electrocardiography of the girl infant at 15 days old, which revealed sinus tachycardia compared with other infants at the same age. Heart rate: 188 beats per minute; RR interval: 339 milliseconds (ms); PR interval: 56 ms; QRS duration: 99 ms; QT interval: 255 ms. [file Image_1.PDF]

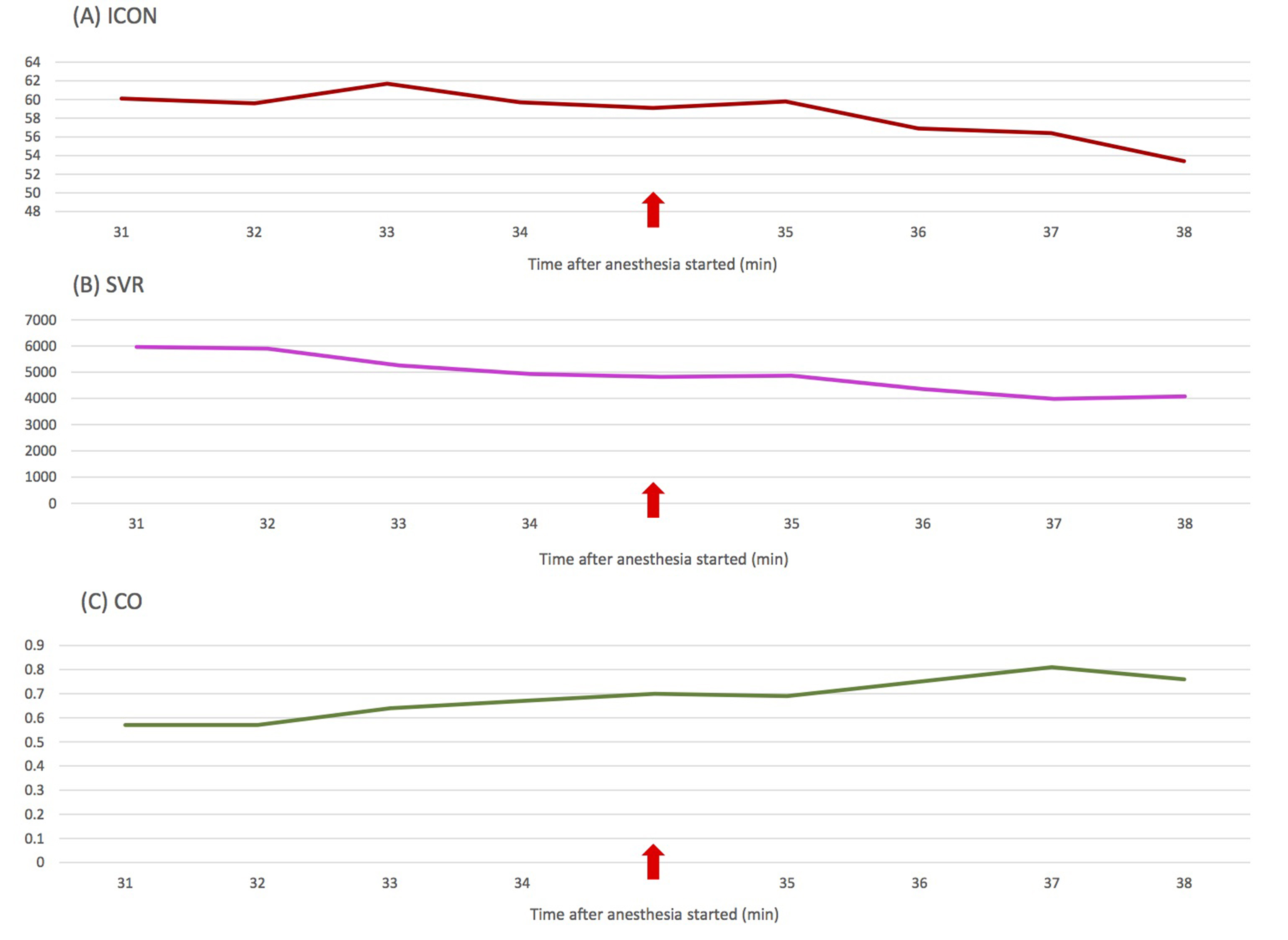

Supplement: Supplementary Figure 2 — Inhalational gas concentration was titrated from 0.7 to 1.0 MAC before skin incision (red arrows indicate the time of titration). ICON and SVR dropped gradually in 1 min, and CO increased. However, ICON and SVR dropped significantly 3 min later. By contrast, CO remained at the same level. MAC, minimal alveolar concentration; ICON, index of contractility; SVR, systemic vascular resistance; CO, cardiac output. [file Image_2.JPEG]
